# Supplementary material for: The Moderation of Perceived Comfort and Relations with Patients in the Relationship between Secure Workplace Attachment and Organizational Citizenship Behaviors in Elderly Facilities Staff
Source: Int J Environ Res Public Health. 2022 Jan 15;19(2):963. doi: 10.3390/ijerph19020963 (PMC8775680; doi:10.3390/ijerph19020963)
Supplement: Supplementary file 1 [file ijerph-19-00963-s001.zip › ijerph-1525180-supplementary.pdf]

APPENDIX 1 - SCALE ITEMS

Secure workplace attachment (Scrima, 2020)

1) I'm attached to my workplace.
2) I would find it very difficult to leave my workplace for good.
3) My workplace is like me.
4) I enjoy the time that I spend in my workplace.
5) I wouldn't enjoy working in another place as much.

Organizational Citizenship Behavior Scale (Podsakoff et al., 1990; Argentero et al., 2008)

Altruism

1) I help others who have heavy workloads.
2) I'm always ready to give a helping hand to those around me.
3) I help others who have been absent.
4) I willingly help others who have work related problems.
5) I take steps to try to avoid problems with other workers.
6) I guide new people even though it is not required.

Civic virtue

7) I keep abreast of changes in the organization.
8) I attend meetings that are not mandatory but are considered important.
9) I attend functions that are not required but help the company image.
10) I read and keep up with organization announcements, memos, and so on.

Conscientiousness

11) I do my job without the need for constant stress from the bosses.
12) I believe in giving an honest day's work for an honest day's pay.
13) I do not take extra breaks.
14) I respect company rules and policies even when no one is watching me.
15) I'm one of most conscientious employees.

Perceived Healthcare Environment Quality Indicators - IUOP (Fornara et al., 2006)

1) (In this in-patient/waiting area) furnishings are in good condition.
2) (In this in-patient/waiting area) the quality of furnishings is good.
3) (In this in-patient/waiting area) walls, floors and ceilings are in poor condition.
4) (In this care unit) one hears noise from outside.
5) (In this care unit) you can hear dins and screams.
6) (In this in-patient/waiting area) the air exchange from outside is adequate.
7) (In this in-patient/waiting area) air humidity is adequate (neither too wet nor too dry).
8) (In this in-patient/waiting area) you can see green spaces from the windows.
9) This in-patient/waiting area has large windows.
10) (This in-patient/waiting area) needs more windows.

11) In this hospital it is easy to find care units and services you are looking for.
12) It is easy to recognize the entrance of this care unit.
13) (In this care unit) you can easily find information points.

Difficult relationship with patients (ambiguous customer expectations of the Customer-Related Social Stressors scale (Dormann & Zapf, 2004)

1) Customers' wishes are often contradictory.
2) It is not clear what customers request from us.
3) It is difficult to make arrangements with customers.
4) Customers' instructions can complicate our work.
5) Some customers ask us to do things they could do by them selves.

Table S1. Results of Exploratory Factor Analysis (EFA) of the Organizational Citizenship Behavior Scale

OCB item	Factor loading		
	1	2	3
Factor 1: Altruism			
20. I willingly help others who have work related problems.	**0.90**		
22. I help others who have heavy work loads.	**0.85**		
16. I'm always ready to give a helping hand to those around me.	**0.82**		
19. I help others who have been absent.	**0.76**		
25. I guide new people even though it is not requied	**0.70**		
Factor 2: Conscientiousness			
5. I respect company rules and policies even when no one is watching me		**0.86**	
8. I do not take extra breaks.		**0.66**	
2. I do my job without the need for constant stress from the bosses.		**0.64**	
Factor 3: Civic Virtue			
7. I attends meetings that are not mandatory, but are considered important.			**0.93**
12. I attends functions that are not required, but help the company image.			**0.88**
15. I keep abreast of changes in the organization.			**0.52**
4. I read and keep up with organization announcements, memos, and so on.			**0.50**

Note. n = 129. The extraction method was principal axis factoring with an oblique (Promax with Kaiser Normalization) rotation. Items with factor loading less than 0.30 have been eliminated.

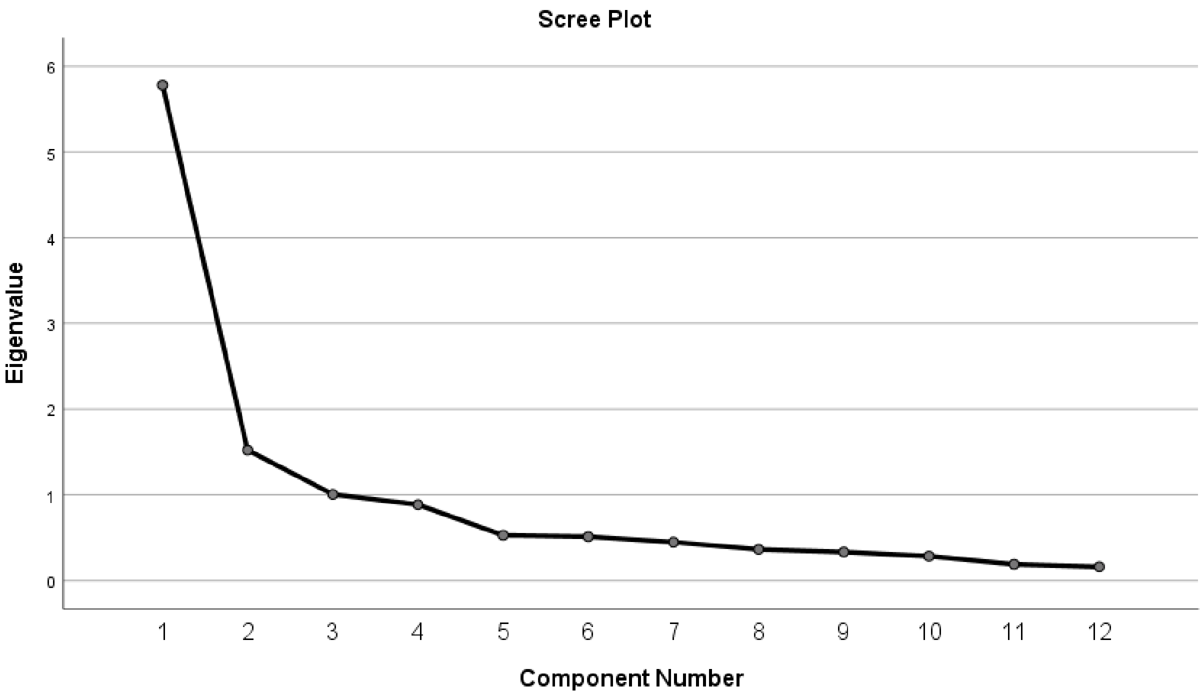

Figure S1. Scree Plot of Exploratory Factor Analysis of the Organizational Citizenship Behavior Scale
